# Inclusivity for people living with disability: the gap between understanding and implementing

**DOI:** 10.1016/j.lansea.2025.100614

**Published:** 2025-06-10

**Authors:** The Lancet Regional Health – Southeast Asia

The *Global report on health equity for persons with disabilities*, published in 2022, estimates that 1.3 billion people have a significant disability. This was followed by the development of the *Disability inclusion guide for action* to guide countries, with their unique contexts, led by ministries of health, engaging with other stakeholders such as other government departments, health service providers, research institutes, and civil society, including people with disabilities and their representative organisations. Last year, the World Health Summit hosted its first-ever session dedicated entirely to health equity for people with disabilities, highlighting the importance of this issue by global health partners.

In southeast Asia, disability prevalence is the third highest in the world, with country prevalence ranging from 3% to 15% of the population. Most countries have instituted laws to safeguard the interest of people with disabilities. India constituted the new Rights of Persons with Disabilities Act 2016, which replaces the old Persons with Disabilities Act 1995. Thailand and Indonesia also have specific laws to promote equal rights, accessibility, and employment for those with disabilities. While these policies mark significant progress, their impact is often limited by gaps in enforcement, social attitudes, resource allocation, and comprehensive planning. Although there is more awareness about disability, there still exists a considerable gap between understanding and inclusion and implementation.

Deep-rooted stigma in societies hinders inclusivity of people with disabilities. Stigma leads to isolation from education, employment, and social participation, and not only presents challenges in identification of disabilities and their diagnosis, treatment, and management, but also reinforces stereotypes and misconceptions, thereby feeding into a cycle of exclusion and inequity. Despite strong policies in place, there have been no efforts to foster social inclusion through education and awareness in the community. While current practice and policies emphasise medical treatment and rehabilitation services, there is a need to provide more attention to the social part of the problem. This could be partly achieved through increasing social participation and building barrier-free environments. Insufficient funding is cited as the main reason for not making public spaces and different environments barrier free. Research consistently shows that interventions focusing on barrier-free environments—such as accessible infrastructure, assistive technologies, and inclusive policies—lead to increased social participation, independence, and quality of life for people with disabilities and their care givers.

As the health landscapes evolve, new challenges are emerging that impact people with disabilities. As society becomes more digital, access to online services, education, health care, and employment increasingly depends on digital literacy and accessible technology. Individuals with disabilities often face barriers to digital access due to lack of accessible websites, digital tools, and assistive technologies. Climate-related events such as floods, cyclones, and heatwaves disproportionately affect vulnerable populations, including people with disabilities. Disasters can lead to injuries, loss of mobility, or separation from support networks, and people with disabilities often face greater barriers in emergency response and recovery. The mental health of people with disabilities is often overlooked as more attention is placed on the physical aspect of the condition. The new mental health policy in Nepal pushes to integrate mental health services into primary care despite significant challenges including governance, funding, and infrastructure. The law recognises mental illness as a disability, but mental health services remain inadequate. For example, mental health facilities are concentrated in urban centres, leaving rural populations underserved.

As more people live longer, there is also an increase in age-related disabilities such as mobility impairments, sensory deficits, and cognitive decline. Older adults with disabilities, particularly women and those living in rural areas, often face compounded barriers in accessing health care, social participation, and support services. In southeast Asia, the rapidly growing elderly population means more people living with disabilities related to ageing, yet social and health-care infrastructure are lacking. In rural Bangladesh, older people reporting cognitive decline have limited access to services and care. These intersections create complex health-care needs, requiring integrated, long-term, and age-friendly support systems.

Use of assistive technology, provision of equal opportunities in education and employment, and barrier-free environments hold the key to promoting inclusivity and equity. However, inability to afford this technology and limited availability are common barriers to their usage. These will require a substantial shift in attitude and policy attention. This cannot be achieved without including people living with disabilities in the decision-making process and policy development. In India, the Rights of Persons with Disabilities Act 2016, the Sarva Shiksha Abhiyan (SSA) programme, and the Inclusive Education for Disabled at Secondary Stage (IEDSS) programme aim to ensure that children with disabilities are able to attend school and that they are provided with the necessary support to fully develop their potential, including training the teachers who teach them. These policies have been able to bring noticeable change in terms of enrolment and retention of students and recognition of specific disabilities over the last decade. Although there is a financial allocation for each child, the timely disbursement of this funding is far from guaranteed, questioning the effectiveness of these programmes. Very often children with disabilities are not well accepted by peers and face abuse and discrimination. Similar conditions continue to hinder their development in the workplace. These policies remain incomplete in realising their potential as long as there is little effort in transforming societal attitudes; without such efforts, the acceptance of individuals with disabilities as integral members of the community and the recognition of their right to a full and healthy life remain insufficient. We need to take a big leap forward in this direction by aligning our priorities and actions in making society inclusive for all.

